# Autologous, free omentum grafts for large, open, distal limb wounds in dogs: Technique and outcome in 10 dogs

**DOI:** 10.1111/vsu.14305

**Published:** 2025-07-09

**Authors:** Jason G. Makar, Wendy I. Baltzer

**Affiliations:** ^1^ Sydney School of Veterinary Science, Faculty of Science The University of Sydney Sydney New South Wales Australia

## Abstract

**Objective:**

To describe the operative technique and outcome of free autologous omentum grafting (OG) for wound reconstruction on the distal limbs of dogs.

**Study design:**

Retrospective case series.

**Animals:**

A total of 10 client‐owned dogs.

**Methods:**

Medical records (2010–2023) were searched for dogs with open limb wounds distal to the stifle or elbow treated with OG for wound reconstruction. OG was harvested via celiotomy, then sutured to the wound bed and 0.4–1 cm subcutaneous tissue beneath surrounding dermis. Wounds were bandaged using a petroleum‐coated primary layer to prevent graft desiccation for 1–2 weeks. Using ImageJ software, wound area and final unhaired scar area were analyzed and time to epithelialization, major and minor complications, and length of follow up were determined.

**Results:**

Dogs with a mean age of 6.9 ± 4.2 years were identified with wounds (*n* = 13 wounds) measuring 25.96 ± 16.27 cm^2^ at the time of OG. Wounds were healed in 59.5 ± 11.1 days. Minor complications included infection and swelling/discharge in two and 10 dogs, respectively. There were no major complications. By 30‐days postoperatively, wounds were 18.85 ± 0.1% of the original size. All wounds healed with complete epithelialization, excellent cosmesis, no lameness and partial to complete hair regrowth.

**Conclusion:**

OG may provide an alternative method for management of distal limb wounds in dogs, with minimal complications, excellent cosmesis and functional outcomes in 10 dogs.

**Clinical significance:**

Free OG may offer an alternative method of wound management in dogs; however, further research with controlled prospective studies is indicated before recommending the method over other treatment options.

**Abbreviations**
NPWT: negative pressure wound therapy
OG: omental grafting
VEGF: vascular endothelial growth factor

## 1 INTRODUCTION

The distal limbs of dogs have minimal skin distensibility and a lack of available skin for primary wound closure especially over high‐motion joints or when large defects are present. There have been many different techniques described to enhance healing and produce an acceptable outcome for full thickness distal limb wounds including free skin grafts, seed skin grafts, and delayed wound closure with or without a graft and negative pressure wound therapy.^1^, ^2^, ^3^, ^4^, ^5^ Second intention wound healing of the distal limb for large cutaneous defects reportedly results in complete healing in approximately 53 days; however, long‐term complications occur in 25% of dogs.^6^

Many surgical grafting techniques rely upon meticulous preparation of the donor site with or without advanced instrumentation, and require minimal movement postoperatively at the recipient bed.^1^, ^2^, ^6^, ^7^, ^8^ Distal limb wound skin graft survival may be up to 90%; however, a higher rate of failure and complications ranging from 38% to 50% in cats and dogs is reported.^1^, ^2^, ^9^, ^10^ The addition of honey or platelet rich plasma may improve skin graft survival from 50% in controls to 67%–83% in treated grafts; however, complications with wound contracture and joint dysfunction remains a potential long‐term problem.^11^ Negative pressure wound therapy (NPWT) or hygroscopic tissue expanders improve skin graft survival in distal limb wounds but require specialized equipment and more challenging aftercare, including the same immobilization challenges faced with full thickness skin grafts alone.^12^, ^13^, ^14^

The omentum is an organ in the abdomen that serves many purposes including its ability to provide lymphatic drainage, blood supply and seal off necrotic or infected abdominal organs.^15^, ^16^, ^17^ It is comprised of two folded sheets of tissue, rich in vascular endothelial growth factor (VEGF) as well as omnipotent stem cells; which allow for angiogenesis and repair of damaged tissue in the abdomen.^16^, ^18^ Omentum is often used in abdominal surgeries to reinforce incisions in viscera especially that of small intestinal anastomoses.^19^ Free autologous OG has been used in various studies to aid in healing fractures, myocardium, wounds and peripheral nerves in humans, cats and dogs.

Omentum may be invaluable to the process of healing of the dermis throughout the wound healing phases including hemostasis, inflammation, proliferation and remodeling. Activated OG provides continuous growth factor stimulation and stem cell precursors to the healing wound bed.^20^ Omentum contains pluripotent stem cells able to differentiate into CD45‐CD34+ subsets, and may differentiate into endodermal, mesodermal and ectodermal lineages including nerve, adipose, hepatic, myocardial, bone, pancreatic and epithelial cells.^21^, ^22^, ^23^, ^24^, ^25^, ^26^, ^27^ Omentum also has myeloid derived suppressor cells which act, along with stem cells, to provide immunomodulatory and anti‐inflammatory properties during the various phases of wound healing.^18^, ^21^

Pedicled autogenous greater omentum has been used as a pedicle flap or microvascular anastomosis to augment free skin graft‐healing of a large wounds in humans, cats and dogs.^28^, ^29^, ^30^, ^31^, ^32^, ^33^ In cats, axillary and trunk wounds receiving pedicled omentum grafts healed with fewer complications, although only cases combined with a skin flap resulted in consistent healing within 14 days.^33^, ^34^, ^35^ Consideration of movement of the pedicle, tension and disruption of neovascularization may contribute to complications of omentum pedicles or anastomoses. Alternatively, free omental grafting does not have these constraints and may improve omentum graft survival, despite a period of ischemia postoperatively.^34^, ^36^, ^37^, ^38^

The purpose of this study was to describe the technique of using OG in large open wounds of the distal limb in dogs. Our hypothesis was that free autogenous omental grafting of large distal limb wounds in dogs results in wound healing without major complications, with excellent cosmesis and function at the time of complete healing.

## 2 MATERIALS AND METHODS

Records of dogs that received OG for distal limb skin defects between January 2010 and May 2023 from the University Veterinary Teaching Hospital Sydney and Massey University were included for analysis. These dogs received OG to manage wounds resulting from either traumatic injury or surgical procedures. All cases were included only if information regarding signalment, histologic diagnosis, postoperative complications, bandage care protocols and long‐term outcome (>50 days postoperatively) were available. Information including wound duration, wound size, culture and sensitivity, and photographs were collected at the time of each bandage change. Bandage changes occurred every 1 to 14 days and varied with as wounds healed with photographs of the wounds taken at every bandage change.

### 2.1 Surgical technique

Traumatic wounds were debrided, lavaged with sterile saline, wound edges freshened to bleeding dermis using sharp dissection, then undermined to expose the underlying subcutaneous layer by 0.5–1.0 cm from the wound edges. As previously described, a 2‐cm laparotomy was performed and 5–50 cm of greater omentum was ligated en bloc using 3‐0 PDS and transected.^39^ The laparotomy was then closed routinely. The free omental graft was placed in sterile saline until mass resection was performed or immediately placed in the wound bed if the wound was traumatic. For surgically created wounds, mass removal included dermis with 2–3.0 cm margins and one fascial plane deep. Resected tissue was marked for orientation, then placed in 10% buffered formalin for histopathologic analysis. OG, in two‐layers, was placed over the wound and tacked approximately every 1.0 cm^2^ using interrupted 3‐0PDS to the deep tissues with care taken to avoid neurovascular structures. OG edges were inserted deep to the dermis 0.5–1.0 cm and secured with interrupted sutures every 1–2.0 cm to the subcutaneous. Any areas of excessive OG motion over the wound were identified using digital palpation, then secured with interrupted suture.

Wounds were covered with 1–2 layers of paraffin‐impregnated gauze (Jelonet, Smith & Nephew), then either an absorbent or silver‐impregnated absorbent layer (Telfa, Covidien or Allevyn, Smith & Nephew, respectively), followed by absorbent layer (Soffban, Smith & Nephew) and finally a tertiary layer (Cohesive bandage, Covetrus). Wounds were inspected daily for 1–2 weeks, then every 3–7 days. Once fully granulated with minimal exudate, bandages were comprised of a single non‐adhesive layer (Telfa, Covidien) and an adhesive bandage (Fixomull stretch, BSN medical) until epithelialized to prevent self‐trauma.

All patients received perioperative prophylactic IV antimicrobials: cefazolin (30 mg/kg 30‐min preoperative, 90‐min perioperative) or other based upon preoperative culture and susceptibility. Perioperative opioid analgesia (methadone 0.2 mg/kg IV every 4 h) and postoperative non‐steroidal anti‐inflammatories (meloxicam 0.1 mg/kg orally every 24 h or carprofen 2.2 mg/kg orally every 24 h) and/or postoperative antimicrobials were administered at attending clinician discretion.

### 2.2 Image analysis

Photographs obtained by smartphone photography were analyzed with ImageJ 1.45 s freeware (National Institutes of Health, Rockville, Maryland; http://imagej.net/ImageJ). A ruler or other instrument placed next to the wound in parallel with the healthy skin was used for image calibration. Wound size, granulation, epithelialization and hair growth were recorded for each timepoint available for each wound.^40^

### 2.3 Owner assessment

Owners were called with a simple questionnaire to assess dogs' lameness (yes or no), owner satisfaction (extremely satisfied, satisfied or unsatisfied) and presence of reoccurrence of the initial lesion in the event of tumor resection.

Results are reported as median and range using descriptive statistics using Excel (Microsoft, Inc) unless otherwise indicated.

## 3 RESULTS

A total of 10 dogs were identified that met the inclusion criteria. Medical records indicated OG was performed as an alternative to secondary wound closure and skin grafting techniques, due to the severity of the wounds, requirement for further surgery with other techniques, presenting comorbidities and potential complications including infection. The omental graft procedure was selected after consideration of the relevant literature, including studies in both human and small animal models. Owners were informed of the risks and potential complications of omental grafting as well as other treatment options for management of distal limb wounds.

There were six male (4 neutered, 2 entire) and four female dogs (all neutered). The breeds were Greyhound (*n* = 2), Boxer (*n* = 2), German Shepherd (*n* = 1), crossbreed (*n* = 1), Golden Retriever (*n* = 1), English Staffordshire Bull terrier (*n* = 1), Rhodesian Ridgeback (*n* = 1), Cavalier King Charles Spaniel (*n* = 1). Mean age of the dogs was 6.8 years (range: 10 months–11.5 years) and bodyweight was 29.6 ± 9.8 kg (mean, SD). A total of 13 wounds were analyzed with cause as: dog bite wounds (*n* = 6), mass resection (*n* = 5), motor vehicle trauma (*n* = 1), and chronic non‐healing wound with multidrug resistant infection (*n* = 1). Mass resection histopathologic diagnosis included: mast cell tumor (*n* = 3), soft tissue sarcoma (*n* = 1), and cutaneous histiocytoma (*n* = 1). All wounds were located in the distal limb (distal to the elbow or stifle) with five wounds at or below the level of the carpus or tarsus (Table 1). The dogs with bite wounds were treated empirically with antimicrobials for 7 to 21 days postoperatively. All celiotomy sites healed without short‐ or long‐term complications noted by the veterinarian or owner. Only Case 1 received culture and susceptibility testing at the time of surgery and Case 3, 2 weeks following surgery after biting at OG (Table 1).

**TABLE 1:** Cases of omental grafting including location, indication, dimensions, time to closure, final function and any complications.

Dog	Age (years)	Location	Indication	Concurrent injury	Wound dimension in (cm^2^)	Time to epithelialize (days)	Final limb function	Culture, antimicrobials administered	Complications	Last assessed (days from surgery)
1	2	LF paw	Wound breakdown	None	7.09	146	No lameness‐vet	Beta‐hemolytic Streptococcus; *Escherichia coli*; chloramphenicol 1500 mg orally every 8 h for 3 weeks	Discharge	171
2	11	LF paw	Soft tissue sarcoma	None	6.47	64	No lameness‐vet	Intraoperative cefazolin 22 mg/kg IV every 2 h	Discharge	759
3	9	RF antebrachium	Dog bite	RF carpus intra‐articular bite wound – joint lavaged concurrently with graft placement	22.9	61	No lameness‐vet	Enterococcus faecalis; amoxicillin clavulonate 20 mg/kg orally 12 h for 5 days	Discharge	61
4	11.5	RF antebrachium	Mast cell tumor	None	34.35	106	No lameness‐owner	Intraoperative cefazolin 22 mg/kg IV every 2 h	Discharge	335
5	5	RH tarsus and pez	Degloving injury	Comminuted fracture of the tibia and fourth metatarsal with external skeletal fixator	28.16	96	No lameness‐vet	Intraoperative amoxicillin clavulonate 20 mg/kg IV every 2 h then orally 12 h for 3 weeks	Discharge	96
6	0.83	LF Antebrachium	Mast cell tumor	None	23.8	69	No lameness‐vet	Intraoperative cefazolin 22 mg/kg IV every 2 h	Discharge	540
7	5	LF antebrachium	Mast cell tumor	None	32.4	101	No lameness‐vet	Intraoperative cefazolin 22 mg/kg IV every 2 h	Discharge	101
8	10.5	LH mid‐tibial	Dog bite	Soft tissue injuries	48.7	75	No lameness‐vet	Intraoperative cefazolin 22 mg/kg IV every 2 h	Discharge	280
9a	5.5	RF distal antebrachium	Dog bite	Soft tissue injuries, sepsis	45.58	67	No lameness‐vet	Intraoperative amoxicillin clavulonate 20 mg/kg IV every 2 h then orally 12 h for 3 weeks	Discharge	270
9b		RF proximal antebrachium	Dog bite	Extensor carpi radialis rupture, sepsis	14.29	50	No lameness‐ vet	As above	Discharge	
9c		LF proximal antebrachium	Dog bite	Sepsis	52.9	67	No lameness‐ vet	As above	Discharge	
9d		LF distal antebrachium	Dog bite	Sepsis	5	39	No lameness‐ vet	As above	Discharge	
10	8	LH plantar metatarsal	Cutaneous histiocytoma	None	15.91	LTFU	No lameness‐ owner	Intraoperative cefazolin 22 mg/kg IV every 2 h	Discharge	57

Abbreviations: h, hours; IV, intravenous; kg, kilograms; LF, left forelimb; LH, left hindlimb; LTFU, lost to follow up; mg, miligrams; RF, right forelimb; RH, right hindlimb; vet, veterinarian.

**FIGURE 1:**
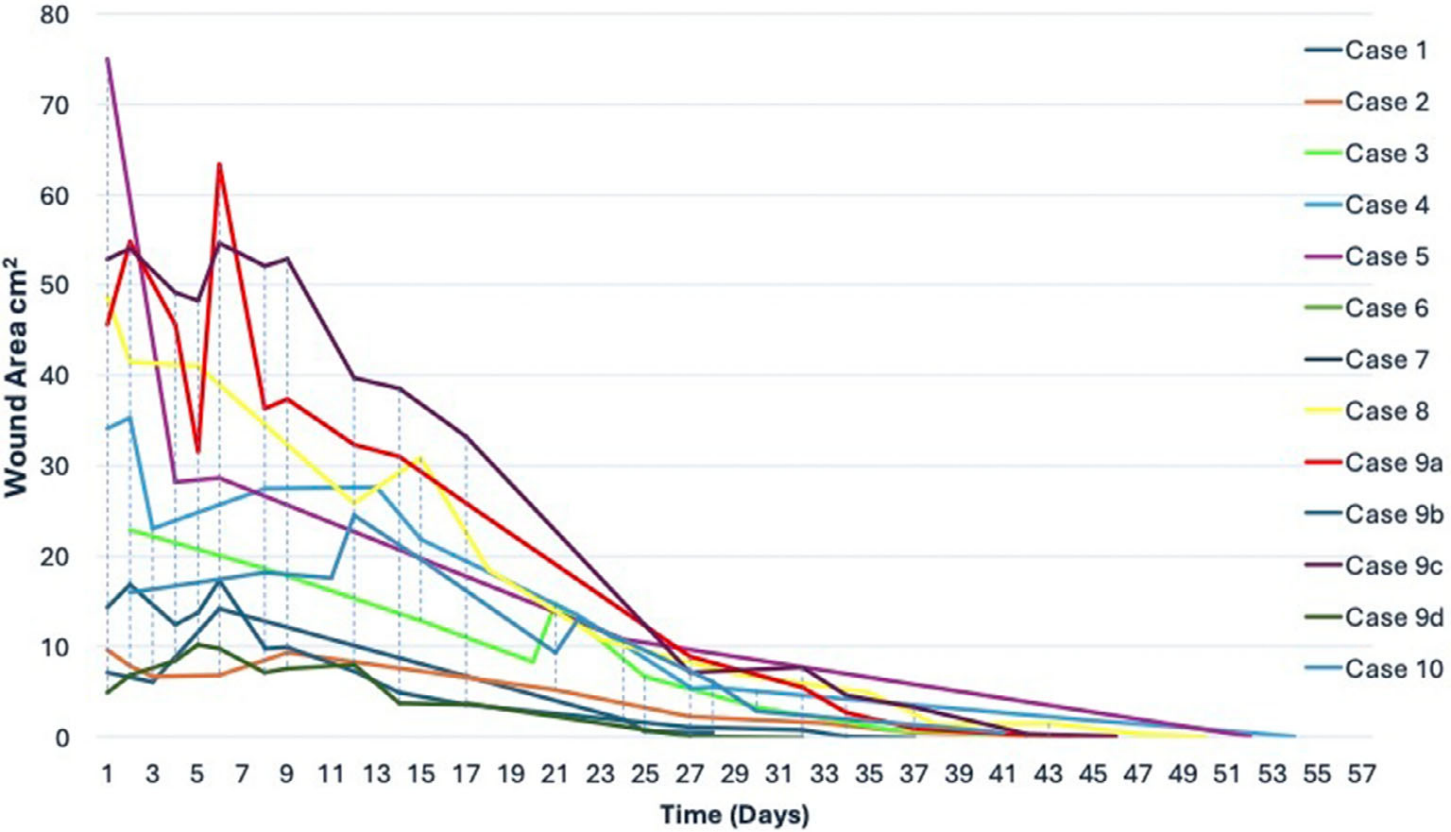
Graph of wound area (cm^2^) for each dog over time (days) determined from available images following free autogenous omental graft for management of distal limb wounds of dogs.

**FIGURE 2:**
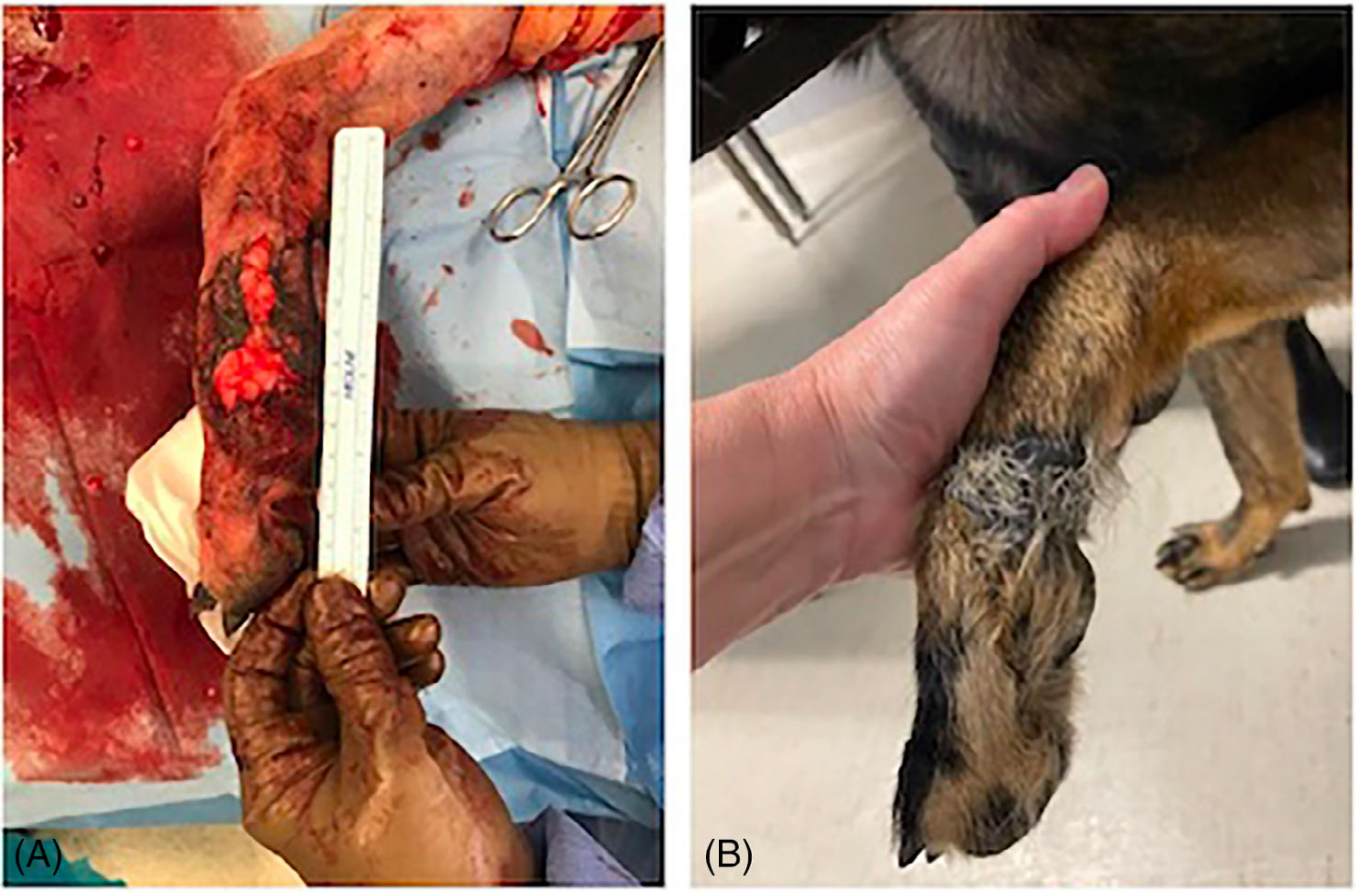
Images of wound in dog 1 located at the lateral metacarpal (digit 5) region of the left forelimb (A) intraoperatively and (B) a total of 176 days postoperatively at final examination. The dog was a police dog with an injury sustained while tracking. Following 3 weeks of wound management at the referring veterinarian, the wound was cultured with susceptibility and omentum grafting. This dog was fully operational without signs of lameness at the time of the final examination. Proximal is to the top and cranial to the left of the images.

Initial wound size was 26 ± 16.3 cm^2^ (mean, SD; range: 4.9–45.8 cm^2^, Figure 1). All wounds included the full‐thickness skin and subcutaneous layer, and in 11/13 wounds, underlying fascia and musculoskeletal tissue were also affected (Figure 2). Establishment of a complete granulation bed (Figure 2) occurred in 3 days (mean 4.7, range: 2–12 days). Wounds had completely epithelialized in 68 days (mean 78.4, range: 36–146); however, multiple wounds had inconsistent follow up (inconsistent follow up was defined as more than 4 weeks between veterinarian assessments). If inconsistently followed dogs were removed from analysis, eight wounds were healed in an average of 59 days (median 65.5, range: 39–75).

### 3.1 Short‐term outcome

**FIGURE 3:**
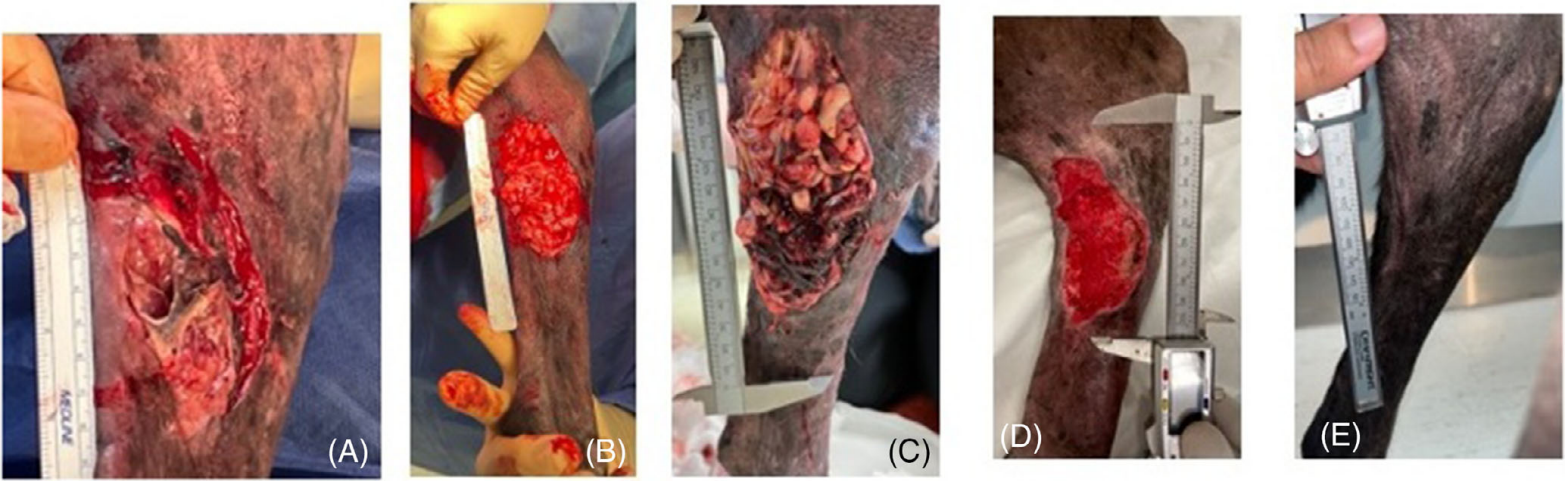
Images of a dog bite wound in dog 8 sustained to the proximal medial aspect of the left tibia (A) immediately preoperatively, (B) following debridement and free omental graft intraoperatively, (C) thirty‐six hours postoperatively, (D) ten days postoperatively, and (E) at final examination 14 weeks postoperatively. Proximal is to the top and cranial to the right of the images.

**FIGURE 4:**
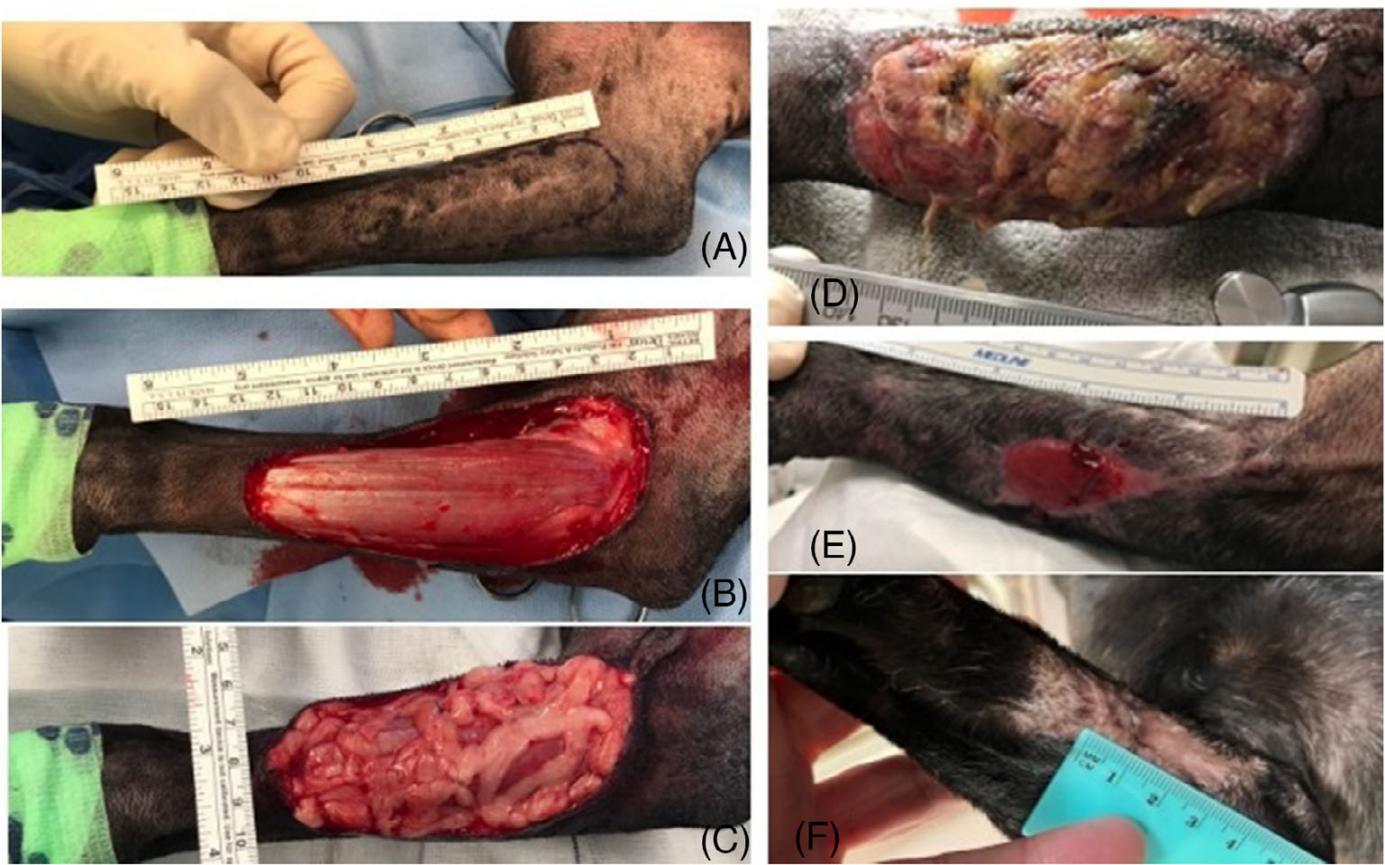
Images of dog 4 presenting for an incomplete resection of a cutaneous mast cell tumor on the medial aspect of the right antebrachium. (A) Immediately preoperative, (B) following resection of surgical scar with 3 cm margins and one fascial plane deep, (C) intraoperative, prior to bandaging, (D) eight days postoperatively, (E) three weeks postoperatively and (F) at final examination 7‐months following omentum grafting surgery. Dorsal is to the top and proximal is to the right of the images.

All dogs underwent daily bandage changes for the first 3–5 days. The time between bandaging was increased according to the amount of discharge produced; and where there was less discharge present, fewer bandage changes were administered. All wounds developed a copious serous to serosanguinous discharge and the superficial layer of omentum became bruised, purple to black in color and markedly swollen (Figures 3, 4 and 6). In some cases, during the bandage change, the wound was lavaged with sterile saline and string‐like strands of superficial omentum were removed along with blood clots and fatty tissue, revealing that the deeper layer of omentum was now a pink bed of granulation tissue. Figure 6D shows the granulation tissue visible beneath the superficial layer of omentum fat. Following the second week, the primary contact layer was changed to a nonadherent, dry primary layer and subsequent bandages were changed every 5–7 days until the wound had completely epithelialized (Figure 2). Enlargement of wound size was identified in several dogs (Figure 1) between 5 and 17 days post‐OG, which resolved within the subsequent 3–7 days.

By one‐month postoperatively, wound area was recorded to be an average of 18.8% ± 0.1% of the original size (Figure 3). At this stage most wounds were small enough to be managed with a small bandage to cover the wound and only weekly assessment of wound size. In some dogs, owners sent in images of the wounds to monitor its healing with rulers placed adjacent and level to, the wound bed. No major or minor complications were noted at the celiotomy site following surgery.

**FIGURE 5:**
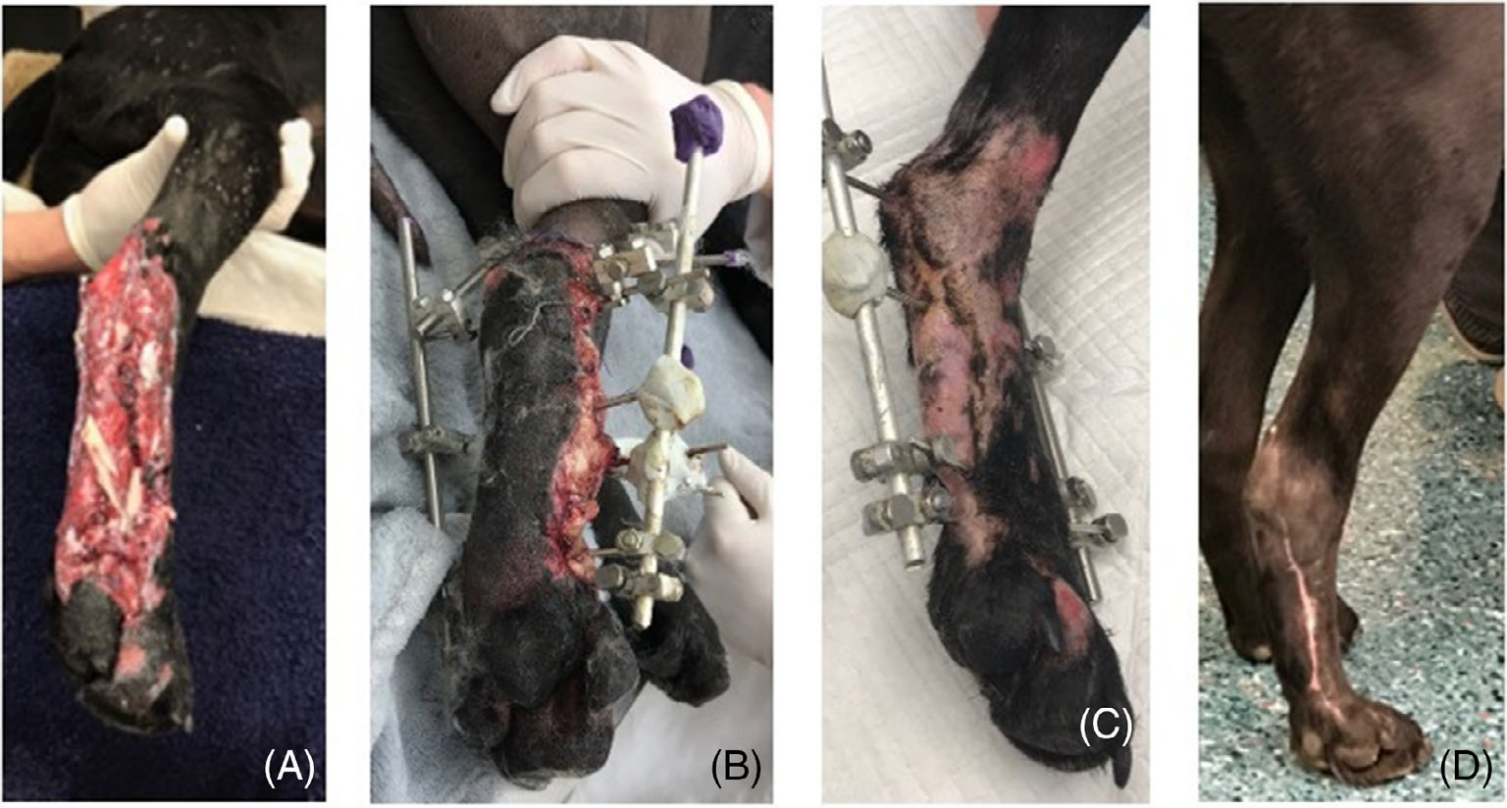
Dog 5 right hindlimb images laterally and distally showing degloving wound following motor vehicle trauma 24 h previously. (A) Preoperatively, (B) twenty‐four hours postoperatively, (C) twelve weeks postoperatively, immediately prior to fixator removal and (D) a total of 150‐days post‐omentum grafting. Proximal is to the top and cranial is to the right of the images.

Dogs 3 and 5 developed a foul smell from the wound 8–19 days postoperatively (Figure 4) and were administered antimicrobials (amoxicillin clavulonate 20 mg/kg every 12 h for 2 weeks) for a presumptive bacterial infection of the wound; however, a culture was performed only on dog 3 which grew an *Enterococcus faecalis*, susceptible to amoxicillin clavulonate. Dog 5 developed a fracture of the fourth metatarsal bone 12 weeks postoperatively following external skeletal fixator removal, which was treated with a bone plate and screws (Figure 5). Dog 5 healed without further complication (Figure 5E).

### 3.2 Long‐term outcome

**FIGURE 6:**
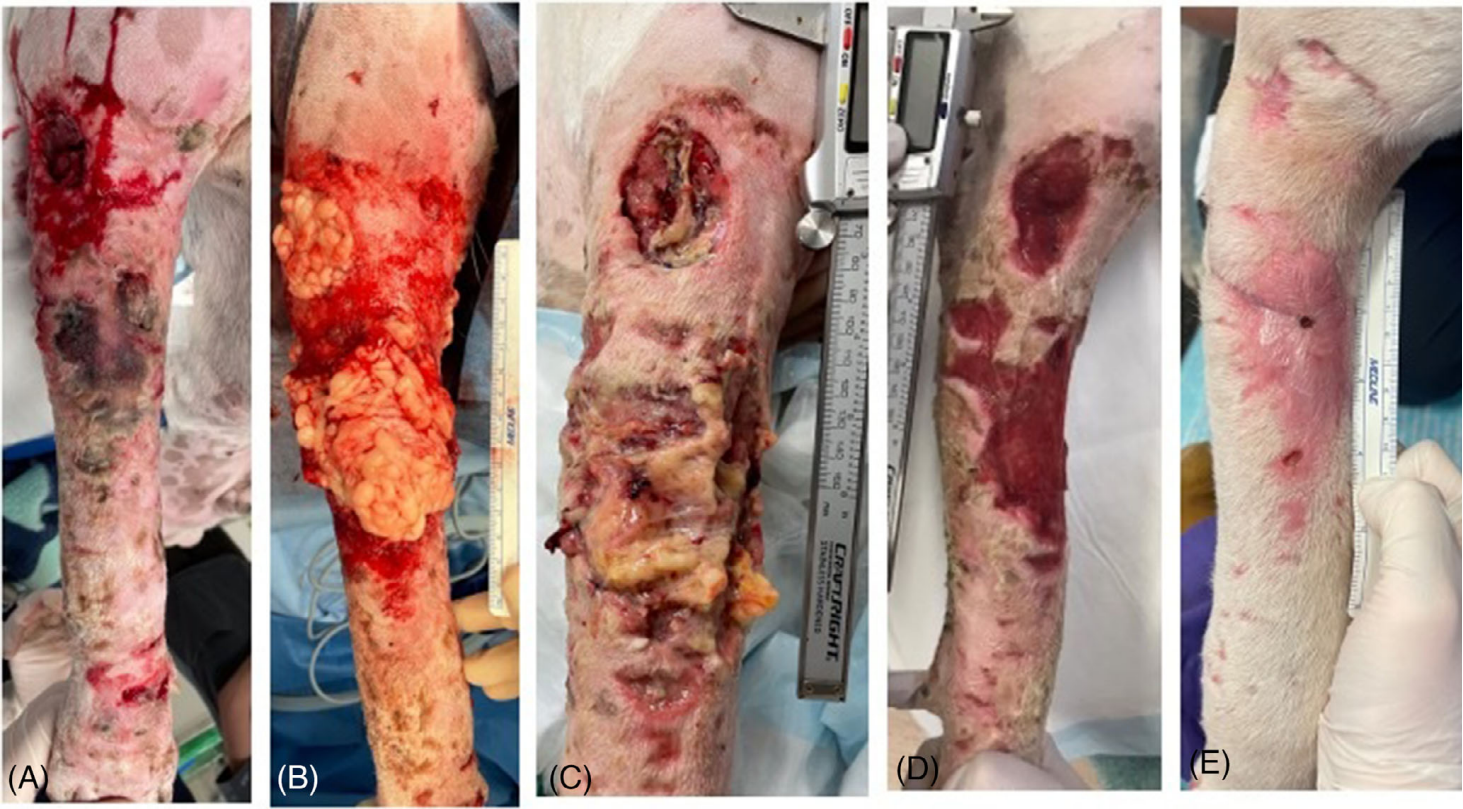
Images of the dorsolateral aspect of the right forelimb of dog 9. The dog presented 5 days following bite wounds to bilateral forelimbs and was unable to extend the right carpus and knuckling over when trying to walk on the limb. (A) Immediately preoperative with most proximal wound involving rupture of the extensor carpi radialis muscle, (B) intraoperative following wound debridement and omentum grafting, (C) seventy‐two hours, (D) eight days and (E) twenty‐four days postoperatively when the ability to extend the carpus returned and knuckling over at carpus had completely resolved. Proximal is to the top and lateral to the left of the images.

All dogs were ambulating with no owner or veterinarian reported lameness on their operated legs at the time of complete healing and at the time of final assessment (median 270 days; range: 57–759). All wounds had epithelialized and had visible hair growth (Figure 6). This was especially evident in Case 1 (Figure 2) where the new fur growth was markedly longer than that of the surrounding fur of the paw and contralateral limb. The size of epithelium remaining unhaired varied between 0 to 20.6 cm^2^ (median: 2.2 cm^2^) which is a median of 17% of the original wound size (range: 0%–45.6%). Dog 9 (Figure 6) had the largest unhaired epithelialized remaining dermis (45.6%).

### 3.3 Owner assessment of outcome

Cases 1, 4, 5, 7 and 10 had inconsistent follow‐up, and the exact date of complete epithelialization was unknown, the date at which it was finally assessed by a veterinarian was recorded and used as the date of complete epithelialization. Case 10 was lost to follow‐up prior to final healing and therefore was not included in the final calculations of time to complete wound healing. Five owners responded (Cases 1, 2, 4, 6 and 8) to the telephone questionnaire and all five reported no lameness, being extremely satisfied with the outcome in 418 days (range: 176–759). No recurrence of the initial mass was reported in 335 days (range: 176–759) in the four dogs with malignant neoplasia.

## 4 DISCUSSION

OG may be a viable alternative to other forms of grafting techniques without the need for advanced surgical skills, instrumentation, or prior preparation of the wound. OG in these 10 dogs resulted in reliable healing, excellent functional outcome and owner satisfaction without adverse effects to healing or final limb function. OG has been used to heal large skin defects of the dorsum of dogs and large non‐healing wounds in cats, as an alternative to traditional full‐ or partial thickness skin grafting.^33^, ^34^, ^37^ To the authors' knowledge, this is the first report of OG for management of distal limb wounds in dogs. All wounds reported in this retrospective completely epithelialized without major complications requiring subsequent surgical procedures, tissue contracture leading to reduced function, inclusion cysts or any visible lameness.

Previously reported methods of distal limb wound management include open wound management with second intention healing, skin grafting, and delayed wound closure. Second intention wound healing in the distal limb has an acceptable outcome in most dogs; however, the epidermis is unhaired, epidermal disruption occurs in 16% long‐term, and contractures with reduced limb function occur in 9.7% of cases.^6^ Skin seed‐grafting following granulation of the wound bed produces a more functional outcome than second intention healing alone in distal limb wounds, but requires a second surgical procedure and may result in epidermal inclusion cysts requiring a third surgery.^3^ OG has been previously reported to establish a new blood supply and stimulate healing in long bone fractures which indicated it may have a role in other traumatized tissue repair.^36^, ^39^

Omentum grafting using microvascular anastomosis has been reported previously in humans and experimentally in dogs for distal limb wounds sustained following trauma or dog bites with good to excellent results.^28^, ^30^, ^41^, ^42^, ^43^ The technique of OG in humans differs from this report in that microvascular anastomosis is performed and the omentum is not grafted to subcutaneous tissue beneath the epidermis at the wound edges.^30^, ^32^, ^41^ Microvascular anastomosis of omentum to limb wounds in dogs resulted in establishment of blood flow communication between the wound bed and omentum in 7 days, confirming omentum neovascularization occurs in canine limb wounds regardless of whether the microvascular anastomosis remained patent.^44^ Omentum was not inserted beneath the skin along the wound edges in this study, either, which may have affected the graft's neovascularization abilities. OG without anastomosis may have more rapid neovascularization if placed in contact with the subcutaneous tissue surrounding the wound but requires further study. OG placed in the subcutaneous tissue of rabbits becomes vascularized by 7 days post‐grafting, with significantly greater neovascularization compared to controls at 28 days.^38^ If microvascular anastomosis is not required, OG for wound management would be more accessible since specialized training and equipment are not needed.

Distal limb full thickness skin grafting complication rates may be up to 66%, and graft success in 38% of dogs.^2^ Skin graft survival increases with Nigeria bee honey, platelet rich plasma or NPWT devices (66%, 50% and 87.5%–100%, respectively), in part, due to reduced infection of the grafts.^11^, ^13^, ^14^ In humans, omentum flaps for infected wounds and joints is advocated due to its profound antimicrobial actions, and OG without anastomosis in infected wounds reported here also resulted in complete healing.^30^, ^32^, ^45^ Contraindications for skin grafting include the presence of a bacterial infection and/or reduced soft tissue coverage of the recipient bed, or large chemical or thermal wounds to large areas of the body.^1^, ^2^, ^9^, ^10^ Infection has been reported in axial pattern flaps and full thickness skin grafts to potentially cause failure of the graft site in 30% and 6.3% of cases, respectively.^2^, ^46^ There are high numbers of white blood cells in omental tissue and its increased neovascularization potential may allow for a hastened immune response aiding infection resolution without graft failure.^37^, ^43^, ^47^

In comparison to full thickness meshed skin grafts with the addition of NPWT, OG was relatively simple, robust, and withstood full limb weight‐bearing without external coaptation. OG achieved successful outcomes similar to combined skin grafting with NPWT but without specialized equipment and reduced limb use in order to maintain negative pressure.^8^, ^36^ Combined skin graft with NPWT reduces the number of bandage changes required in the early stages of wound management and is an advantage over OG.^8^, ^13^, ^14^ OG requires frequent bandage changes due to profuse discharge from the wound in the first week postoperatively. The omentum's higher concentration of VEGF which allows inosculation to occur rapidly, may explain this short‐term, postoperative complication.^21^

Wound size increased following OG in most dogs between days 5 and 17, then the size consistently decreased thereafter (Figure 1). Similarly in cats free grafted with OG, wound size increased between 3 and 15 days postoperatively.^37^ Conversely, OG as an adjunct to internal fracture repair results in limb swelling and discharge for only 3 to 5 days.^36^, ^39^ The difference in open wound and closed fracture swelling, discharge and limb size may be due to the soft tissue envelope constraining fractures following surgery or neovascularization of wounds may require more time than OG in a closed fracture environment. Omentum is able to produce neovascularization and tissue healing at a rapid rate due to its ability to produce vascular endothelial growth factor (VEGF) and basic fibroblastic growth factor (b‐FGF) from specialized omental microvascular endothelial cells, omentum‐specific fat cell production of angiogenic lipid soluble factor, and specialized omentum stem cell rapid reproduction.^48^, ^49^, ^50^ Milky spots in activated omentum increase stem cells and other vasoactive cells from occupying 7% of omentum to 76%.^51^ While limb and wound swelling and discharge are remarkable, management with frequent bandage changes was effective and was considered a minor complication of this technique and similar to short‐term complications associated with distal limb second intention wound healing methods.^6^

Of the eight wounds that had consistently recorded follow‐up, mean healing time was 59 days and is comparable to the healing time of distal limb wounds managed by second intention healing following soft tissue sarcoma removal.^6^ Second intention healing results in 93.5% of dogs requiring no further surgical intervention and complete epithelialization in approximately 53 days.^6^ Comparably, complete epithelialization occurred in 100% of OG dogs in approximately 59 days. However, in the study of second intention healing of distal limb wounds, 25% of patients experienced long‐term complications, including epidermal disruption, reduced range of motion due to wound contracture, and persistent lameness.^6^ These complications were not observed in the current study. The smaller sample size and shorter follow up (median 270 days; range: 57–759) may have resulted in fewer long‐term complications; however, all 10 dogs had an absence of wound contracture or scar adherence to fascia following complete healing. The follow‐up period in the aforementioned study was a median of 980 days. The disparity in healing times and complications between the two wound management techniques warrants further investigation with longer assessment periods.

The versatility and robustness of OG was evident in its variable use in concurrence with orthopedic injuries requiring external skeletal fixation (Case 5, Table 1), presence of multi drug resistant infection in a chronic wound (case 1, Table 1), in oncologic surgery (Cases 2, 4, 6, 7, 10, Table 1), and multiple wounds in one dog with systemic sepsis (Case 9a–d, Table 1) where primary closure was impossible and skin graft use was contraindicated. The greater omentum is readily available for use to cover large defects in cases of large septic wounds, and along with the presence of immunomodulatory cells and macrophages, it aids in the resistance and treatment of bacterial infections.^15^, ^16^, ^18^, ^34^ Two wounds (Case 3 and 5) had malodorous discharge in the follow‐up period, which were treated with antibiotics and resolved with no further treatment. However, wounds were not routinely cultured throughout the healing process, which is a significant limitation. Postoperative antibiotic therapy was administered in cases with a known infection based on prior culture (Case 1, Table 1) and in instances of trauma not treated within 6 h of injury (Cases 3, 5, and 9, Table 1). Of these, two cases had positive cultures (Cases 1 and 3). Notably, none of the oncologic procedures showed any evidence of infection, in contrast to a reported infection rate of four out of 31 cases in a separate study.^6^ Numerous reports of the successful use of OG in chronically infected, open wounds in humans supports the use of OG in infected wounds of dogs.^28^, ^30^, ^31^, ^41^

All cases with oncologic surgery resulted in complete tumor resection with clean margins as reported by board‐certified anatomical pathologist assessment of the tissue. The ability to use OG to heal large cutaneous and subcutaneous wounds created by extensive margin resection may have allowed surgeons to comfortably make aggressive tissue resection decisions. At the time of writing this study, all dogs had no reported local recurrence of their respective neoplasia at long‐term follow‐up with no other reported complications or lameness. In one case (Case 4), chemotherapy was initiated while the wound was incompletely healed; however, this did not affect healing. The data presented here is quite limited and recurrences may have developed later in the affected dogs.

Given the retrospective nature of this case series, there were many limitations. Inconsistent follow up for reassessments of the wounds made it difficult to assess exact time of complete epithelialization. At 1‐month post‐OG wounds were <20% of their original size. At this point many of the wounds may have been candidates for delayed primary closure or augmented with other simple techniques to hasten wound healing such as platelet rich plasma injections or skin grafting.^3^, ^52^ In this study, wound sizes were obtained using ImageJ, a program that can be used to easily obtain the area of images in two dimensions. This has inherent inaccuracies when measuring a surface over a three‐dimensional structure such as the distal limb; however, this method results in accurate wound size determination with excellent inter‐ and intrarater reliability.^40^

The focus of this retrospective report was to describe the surgical technique and outcome in 10 clinical cases. Prospective, randomized and controlled clinical trials are warranted to determine the outcome, healing times and cosmesis, compared to traditional wound management techniques. Inconsistent wound culture and susceptibility between dogs and time points made the effect of potential bacterial contamination difficult to determine and this may have affected the time to wound closure. There were inconsistencies in wound size, location, cause and comorbidities which may have affected the results. In addition, owner assessments were inconsistent and incomplete.

All owners that were able to be contacted were satisfied with the outcome and cosmesis at their last contact. The time to complete healing was similar to second intention wound healing.^6^ Hair regrowth and functional outcome is suggestive that OG may play a supportive role in wound healing; however, due to the small study size and retrospective nature of this report, it cannot be determined whether OG has any advantage over second intention wound healing. Reports of free‐OG to assist in the management of non‐healing wounds in cats or experimentally induced wounds in other species support the use of OG for healing of distal limb wounds.^18^, ^34^, ^35^ To fully understand the role that OG plays in the regrowth of new subcutaneous tissue, epidermis, and hair in distal limb wounds of dogs, further studies are required with histologic assessment during the healing process. Tissue‐specific healing may have been due to the presence of a high concentration of growth factors and omnipotent stem cells which may in turn produce new hair follicles and skin resulting in the reported cosmesis and owner satisfaction of this report.^15^, ^16^, ^18^, ^34^, ^36^ Prospective clinical trials comparing OG to second intention wound healing are needed because similar healing times and short‐term complications are reported; prior to the use of this technique over another method.

## 5 CONCLUSION

Free autogenous OG to skin wounds of the distal extremities of dogs does not require special instrumentation and may augment wound healing, overall cosmesis and limb function with few long‐term complications; however, healing time and short‐term complications were similar to reports of second intention healing in dogs following tumor resection of the distal limbs.^6^

## AUTHOR CONTRIBUTIONS

Makar JG, DVM, BSc, MANZCVS (Surgery): Primary author involved in performing case search and data acquisition from two participating hospitals, interpretation, manuscript preparation and revision. Baltzer WI, DVM, PhD, DACVS (Small Animal), DACVSMR‐Canine, DECVS: Developed the surgical technique, designed the study, data collection, manuscript preparation and contributed to its scientific content. All authors provided a critical review of the manuscript and endorse the final version.

## CONFLICT OF INTEREST STATEMENT

The authors declare no conflicts of interest related to this report.
